# Effects of cerebellar repetitive transcranial magnetic stimulation plus physiotherapy in spinocerebellar ataxias – A randomized clinical trial

**DOI:** 10.1111/cns.14797

**Published:** 2024-06-18

**Authors:** Marcus Grobe‐Einsler, Friederike Bork, Aline Faikus, René Hurlemann, Oliver Kaut

**Affiliations:** ^1^ Department of Neurology University Hospital Bonn Bonn Germany; ^2^ German Center for Neurodegenerative Diseases (DZNE) Bonn Germany; ^3^ Department of Psychiatry, School of Medicine and Health Sciences University of Oldenburg Oldenburg Germany; ^4^ SRH Gesundheitszentrum Bad Wimpfen GmbH Bad Wimpfen Germany

**Keywords:** ataxia, cerebellum, SCA, TMS, transcranial magnetic stimulation

## Abstract

**Background:**

In absence of drug therapy options, standard treatment for spinocerebellar ataxia consists of symptomatic physiotherapy and speech therapy. New therapeutic options are urgently needed. Transcranial magnetic stimulation is a promising therapeutic option, but applicability is limited by lengthy duration of stimulation protocols.

**Methods:**

In this randomized sham controlled clinical trial, patients were assigned to verum (*n* = 15) or sham (*n* = 18) cerebellar transcranial magnetic stimulation. To yield best possible treatment effects, both intervention groups received intensified physiotherapy for the duration of the study.

**Results:**

Ataxia severity was reduced by 1.6 points on the Scale for assessment and Rating of Ataxia among patients in the verum group (*p* < 0.001). Clinical improvement was significantly larger in the verum group, compared to the sham group (*p* < 0.01). The treatment effect was mainly carried by improved appendicular coordination. Patients in the verum group also significantly improved in the 8 Meter Walk Test (*p* < 0.05) and PATA rate (*p* < 0.01).

**Conclusions:**

Cerebellar rTMS ameliorates ataxia severity in patient with spinocerebellar ataxia. Condensing treatment duration to only 5 days without reduction of treatment effects facilitates applicability and therefore broadens availability to larger patient populations.

## Graphical abstract

In absence of drug therapy options for patients with spinocerebellar ataxia, new therapeutic options are urgently needed. In this randomized sham controlled trial, 5 days of cerebellar transcranial magnetic stimulation plus physiotherapy improved ataxia severity. The short duration of the intervention broadens availability to larger patient populations.

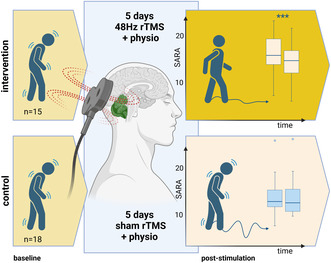

## 1 INTRODUCTION

The disease group of cerebellar ataxias comprises several generally rare diseases with broad pathophysiological and phenotypic heterogeneity. Autosomal dominantly inherited ataxias are referred to as spinocerebellar ataxias (SCAs). Cerebellar atrophy is a shared hallmark of SCAs, resulting in impairment of gait, stance, speech, and coordination.^1^ Additionally, the cerebellum is meanwhile recognized to be involved in the complex processes memory, language, perception, and emotion.^2^, ^3^ Motor symptoms in ataxia are typically assessed by the Scale for Assessment and rating of ataxia (SARA).^4^

Despite first clinical trials on innovative drug therapies for certain SCAs, approved causal or disease‐modifying therapies are still lacking. Symptomatic therapies include physiotherapy and speech therapy, the benefits of which have been proven in studies before.^5^, ^6^, ^7^, ^8^, ^9^ In the absence of drug therapy options, non‐invasive brain‐stimulation (NIBS) is an exciting and promising therapeutic option with a good safety profile.^10^, ^11^ Using transcranial magnetic stimulation (TMS), the cerebellar output can be modified indirectly by stimulating or inhibiting the Purkinje cells in the cerebellar cortex,^12^, ^13^, ^14^, ^15^ particularity lobules VI–VIII.^14^, ^15^ This effect can be prolonged significantly using repetitive TMS pulses (rTMS).^14^, ^16^ In the past years, a number of studies investigated the effects of cerebellar rTMS on ataxic patients with compelling evidence for, at least temporary, positive influence on the disease course.^17^, ^18^, ^19^, ^20^, ^21^, ^22^ Interestingly, stimulatory and inhibitory cerebellar TMS protocols have been tested in cerebellar ataxias, both with reported positive effects on motor function.^11^, ^23^ The stimulation protocols used (stimulation frequency and duration), the number of patients and the study design are, however, very heterogeneous. The duration of most stimulation protocols lay between 2 and 4 weeks. Taking into account the rarity of the disease with a consecutively large areas covered by the involved clinics (meaning long journeys for patients) and patients motor impairment, such long stimulation protocols represent a relevant burden for the patients and are an obstacle for widespread use in clinic. The aim of our study is to test the effects of an accelerated, short stimulation protocol encompassing only 5 days of rTMS targeted to the cerebellum to alleviate ataxia severity in SCA patients. To booster the clinical effect we applied a combination of rTMS and physiotherapy.

## 2 METHODS

All patients were recruited from the Department of Neurology, University Hospital Bonn, Bonn, Germany. All enrolled subjects gave written informed consent for participation. The study was performed in accordance with The Code of Ethics of the World Medical Association (Declaration of Helsinki) and approved by the Ethics committee of the University hospital of Bonn, Germany (No. 208/20, date 11/06/2020). This trial was registered at the German Clinical Trial Registry (DRKS‐ID: DRKS00023473).

Inclusion criteria were age 18 years or older, genetically confirmed diagnosis of SCA, and stable oral medication and symptomatic therapy for at least 1 month. Exclusion criteria were history of seizures, severe head injury or neurosurgical intervention, dementia, the presence of metallic particles or implants in the head, and cardiac pacemakers or neuro‐stimulators.

### 2.1 Study design

The study design was double‐blind, randomized and sham‐controlled. The primary clinical outcome measure was change from baseline score on the Scale for Assessment and rating of Ataxia (SARA).

Participants were randomized to either verum or sham TMS treatment using a block randomization with an AAABBB distribution model. Additional to verum or sham TMS stimulation, patients in both intervention groups received intensified symptomatic physiotherapy according to a standardized multi‐complex therapy scheme.

### 2.2 MRI‐based neuro‐navigation

A neuro‐navigation system (Brain Science Tools BV, De Bilt, Netherlands) was used to achieve optimal coil positioning to the location of the cerebellum.^24^ The region of interest was determined on a structural T1‐weighted magnetic resonance imaging (MRI). The anatomical scan was transformed to a 3D rendered image of skin surface on which the area of interest and craniotopic landmarks were marked. The landmarks were measured directly on the patient's head with a 3D digitiser pen. Finally, the location on the scalp overlying the cerebellar cortex was found by stereotactic navigation.

### 2.3 Clinical assessments

Study participants were assessed at baseline (V0), 3–4 h after completion of the last rTMS treatment at day 5 (V1), and 1 month after the last stimulation (V2). To reduce intra‐ and inter‐rater variability, all investigators that were involved in the clinical assessments (MGE and FB) were SARA trained and certified.^25^ Other clinical assessments included the 8 Meter Walk Test (8MW), the Nine‐Hole Peg Test (NHPT), PATA rate, and the Cerebellar Cognitive Affective Syndrome Scale (CCAS). Computerized dynamic posturography was performend using a standardized balance perturbation method (PosturoMed CMS10; zebrisMedical GmbH, Isny im Allgäu, Germany). Higher values correspond to greater sway, meaning more severe impairment of postural stability.

### 2.4 Intervention

A Magstim Rapid^2^ (Magstim Co. Ltd, Whitland, UK. Device number P/N: 3012‐00) with an air‐cooled figure‐of‐eight coil delivered stimuli with the coil held tangentially over three regions: vermis (over the inion), bilateral cerebellar hemispheres (4 cm lateral to the left of the inion, 4 cm lateral to the right of the inion) (Figure S1). Placebo treatment (sham) was performed with a placebo coil (Magstim) with identical appearance, sound emission, and stimulation of only superficial tissue (muscles). During stimulation, patients laid their head down on a pillow on a small table in front of them. Due to technical specifications of the used coil the stimulation frequency was reduced to 48 Hz, in contrast to standard >50 Hz intermittend theta burst stimulation (iTBS) protocols. The stimulation protocol consisted of 15 sessions (three sessions per day, applied hourly) over 5 consecutive days, resulting in 64,800 pulses per patient. Stimulation was delivered using a pattern similar to a standard intermittent theta burst stimulation (iTBS) paradigm, but with slight modifications. At each stimulation site, pulses were delivered in 48 Hz triplet bursts, 5 bursts per second, in trains of 3.0 s on and 5.4 off, for 32 trains, totalling 1440 pulses per site.

Standard TMS protocols use the motor threshold over the motor cortex (M1) to determine an individual TMS power. However, there is no evidence that stimulation power over M1 correlates with success of cerebellar stimulation. We therefore selected a fixed stimulator output intensity of 50% for cerebellar stimulation in all patients.

### 2.5 Statistical analyses

Intragroup comparisons were performed using paired two‐sample *t*‐tests (SARA and PATA rate) and Wilcoxon‐signed rank test with continuity correction (NHPT, 8MW, Posturomed). Inter‐group comparisons were performed using unpaired two‐sample *t*‐test and Wilcoxon rank sum test, accordingly. *p*‐values were corrected for multiple testing using Bonferroni correction. Effect size for parametric data was calculated as Cohen's *d*. An effect size of Cohen's *d* < 0.5 is considered as small, <0.8 as moderate and ≥0.8 as large. For non‐parametric data, the Wilcoxon effect size *r* was calculated as *Z*‐statistic divided by square root of the sample size. An effect size *r* < 0.3 is considered as small, <0.5 as moderate, and ≥0.5 as large. Statistical significance was defined as *p* < 0.05. All statistical analyses were performed using R (version 4.3.0, R Core Team, 2023).

## 3 RESULTS

Totally 33 patients were included in this study. Totally 15 patients were assigned to the verum group and 18 to the sham group. Mean age was 48.7 years (with sd 13.8) in the verum group and 48.2 years (with sd 13.7) in the sham group. Ataxia severity was higher (but not significant) in the verum group with a mean of 15.8 SARA points (with sd 4.2) in the verum group and 13.6 SARA points (with sd 3.8) in the sham group. The sham group had more female patients (67%) than the verum group (20%). Cognitive function as measured by CCAS was comparable in both groups with a mean score of 2.5 points (with sd 2.3) in the sham group and 3.2 points (with sd 2.2) in the verum group. The diagnoses included 14 patients with SCA 1 (8 in sham), 6 patients with SCA2 (6 in sham), 5 patients with SCA3 (1 in sham), 1 patient with SCA5 (0 in sham), 5 patients with SCA6 (2 in sham), 1 patient with SCA7 (1 in sham), and 1 patient with SCA28 (0 in sham). All randomized patients completed the stimulation protocol and assessments at V0 and V1. Totally 13 patients in the sham group and 10 patients in the verum group were lost for follow‐up (V2), so robust statistical analysis was only feasible for differences between V0 and V1. The study flow‐chart is shown in Figure S2. Adverse events were generally rare and included painful muscle twitches during stimulation (6×), headache after stimulation (1×) and claustrophobia during stimulation (1×). Baseline characteristics and results from assessments at V0 and V1 are summarized in Table 1.

**TABLE 1:** Baseline characteristics (V0) and results from assessments after 5 days of verum or sham rTMS treatment (V1).

	Group	V0	V1
Age	Verum	48.7 (13.8)	
	Sham	48.2 (13.7)	
Gender	Verum	20%	
	Sham	67%	
CCAS	Verum	3.2 (2.2)	
	Sham	2.5 (2.3)	
SARA	Verum	15.8 (4.2)	14.2 (4.1)
	Sham	13.6 (3.8)	13.30 (4.0)
SARA trunc	Verum	9.1 (2.5)	8.3 (2.6)
	Sham	7.8 (2.8)	7.3 (2.8)
SARA app	Verum	6.7 (2.5)	5.9 (2.0)
	Sham	5.7 (1.6)	5.9 (1.6)
NHPT	Verum	58.7 (44.6; 78.0)	55.0 (46.2; 79.6)
	Sham	46.6 (39.0; 52.2)	41.5 (36.5; 47.7)
8MW	Verum	6.3 (5.6; 9.1)	6.0 (4.7; 7.9)
	Sham	6.2 (5.3; 7.4)	6.0 (5.1; 7.0)
PATA	Verum	20.7 (6.2)	24.1 (5.6)
	Sham	21.4 (5.6)	22.6 (6.1)
Posturo	Verum	224.8 (191.7; 404.3)	257.5 (210.0; 290.6)
	Sham	305.0 (245.9; 337.3)	276.5 (234.7; 342.6)
Diagnoses	Verum	6xSCA1, 4xSCA3, 1xSCA5, 3xSCA6, 1xSCA28	
	Sham	14xSCA1, 6xSCA2, 5xSCA3, 1xSCA5m 5xSCA6, 1xSCA7, 1xSCA28	

*Note*: The table provides values as mean with standard deviation for parametric data or median with 25% and 75% quartiles for non‐parametric data.

Abbreviations: 8MW, 8 Meter Walk Test; CCAS, Cognitive Affective Syndrome Scale; NHPT, Nine Hole Peg Test (mean from both hands); PATA, PATA rate; Posturo, Dynamic posturography; SARA app, SARA subscore for appendicular items; SARA trunc, SARA subscore for trunc items; SARA, Scale for Assessment and Rating of Ataxia; SCA, Spinocerebellar ataxia.

**FIGURE 1:**
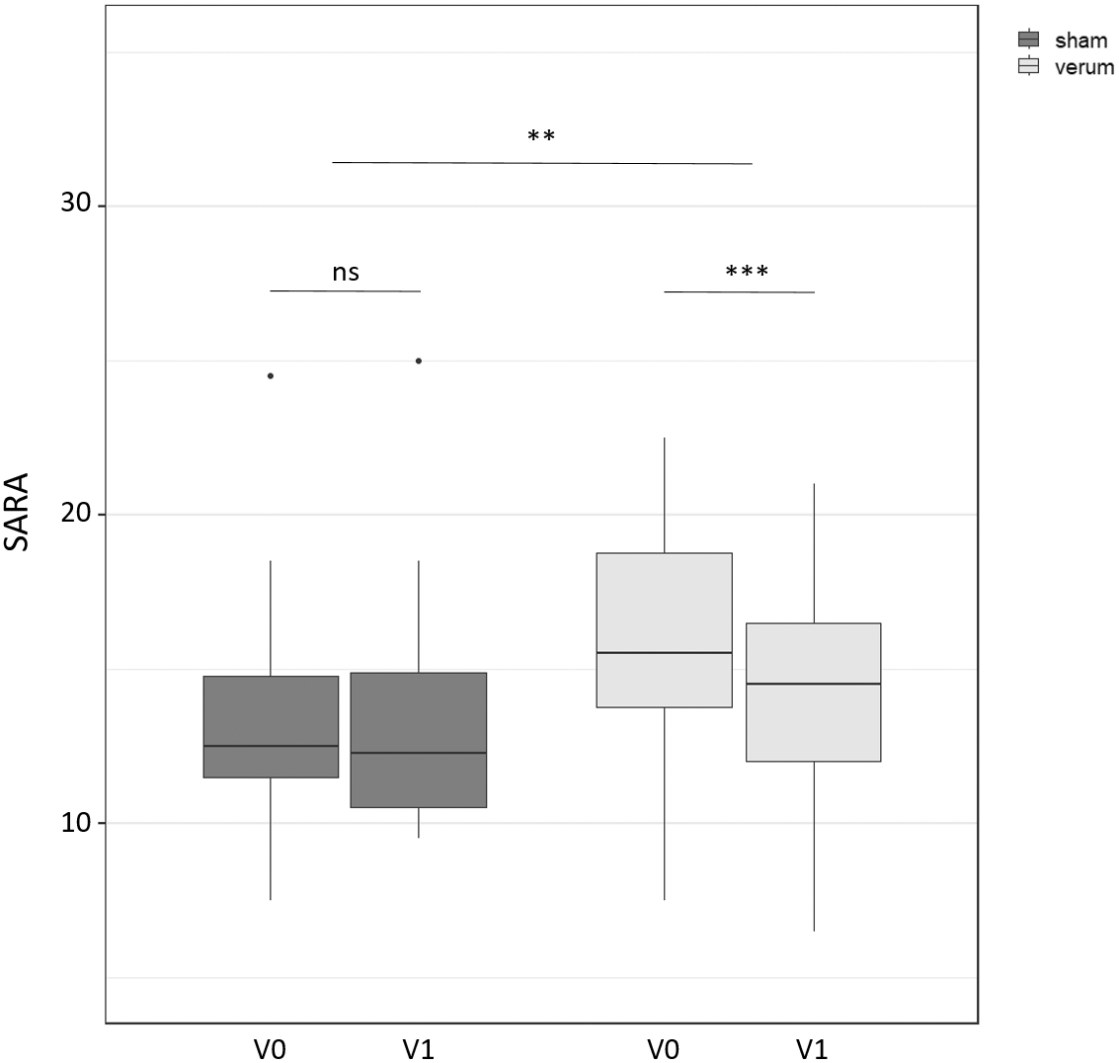
SARA scores from verum and sham group at baseline (V0) and after 5 days of rTMS (V1). Stars indicate the level of significance from Bonferroni adjusted *p* values (**p < 0.01, and ***p < 0.001).

rTMS led to significant improvement in total SARA score between V0 and V1 in the verum group (*p*_adj_ < 0.001, Cohen's *d* = 1.54), but not in the sham group (*p*_adj_ = 1, Cohen's *d* = 0.28). The improvement in the verum group was significant in an inter‐group comparison using unpaired *t*‐test (*p*_adj_ < 0.01, Cohen's *d* = 1.31, Figure 1). Results from intragroup and inter‐group comparisons are summarized in Table 2 for total SARA, SARA subscores and PATA rate and in Table 3 for NHPT, 8MW and NHPT. To investigate which ataxia domains contributed most to the reduction of total SARA, we divided the scale into two subscores: the trunc subscore incorporated SARA items 1–4 (gait, stance, sitting, and speech) and had a range from 0 to 24 points. The appendicular subscore incorporated items 5–8 (nose finger test, finger chase, fast alternating hand movements and heel shin slide) and had a range from 0 to 16 points.

**TABLE 2:** *t*‐Statistics and effect size (Cohen's *d*) of outcome measures with parametric data.

	*T* statistics				Effect size
	*p*_adj_	*t*	*df*	95% CI	Cohen's *d*
SARA					
Intragroup (verum)	<0.001	5.97	14	1.05; 2.22	1.54
Intragroup (sham)	1	1.17	17	−0.22; 0.78	0.28
Intergroup	<0.01	−3.75	29.27	−2.10; −0.62	1.31
SARA trunc					
Intragroup (verum)	0.122	2.70	14	0.18; 1.56	0.70
Intragroup (sham)	0.106	2.67	17	0.11: 0.90	0.64
Intergroup	1	−0.99	22.76	−1.14; 0.40	0.35
SARA app					
Intragroup (verum)	<0.01	4.22	14	0.38; 1.16	1.09
Intragroup (sham)	1	−1.19	17	−0.62; 0.17	0.28
Intergroup	<0.01	−3.80	30.84	−1.52; −0.46	1.32
PATA					
Intragroup (verum)	<0.01	−5.08	14	−4.84; −1.96	1.31
Intragroup (sham)	0.131	−2.60	17	−2.21; 0.02	0.61
Intergroup	0.092	2.66	26.0	0.50; 3.86	0.94

*Note*: Intragroup: Change of outcome measure within a group between baseline visit and after intervention. Inter‐group: comparison of change of outcome measures between sham and verum group. *p*‐values are reported after Bonferroni correction for multiple testing.

Abbreviations: PATA, PATA rate; SARA app, SARA subscore for appendicular items; SARA trunc, SARA subscore for trunc items; SARA, Scale for Assessment and rating of Ataxia.

**TABLE 3:** Test statistics for Wilcoxon signed‐rank test and effect size.

	Wilcoxon tests		Effect size
	*p*_adj_	95% CI	*r*
NHPT			
Intragroup (verum)	1	−3.97; 6.19	0.07
Intragroup (sham)	0.316	0.03; 4.56	0.48
Intergroup	1	−1.93; 5.37	0.16
8MW			
Intragroup (verum)	<0.05	0.25; 1.32	0.76
Intragroup (sham)	1	−0.15; 0.67	0.26
Intergroup	0.251	−1.15; −0.05	0.37
Posturo			
Intragroup (verum)	1	−48.83; 66.50	0.04
Intragroup (sham)	1	−29.67; 34.17	0.07
Intergroup	1	−50.33; 53.67	0.06

*Note*: Intra‐group: change of outcome measure within a group between baseline visit and after intervention. Inter‐group: comparison of change of outcome measures between sham and verum group. *p* values from Wilcoxon‐signed rank test are reported after Bonferroni correction for multiple testing.

Abbreviations: 8MW, 8 Meter Walk Test; NHPT, Nine Hole Peg Test (mean from both hands); Posturo, Dynamic posturography.

Patients showed a significant reduction of the appendicular subscore between V0 and V1 in the verum group (*p*_adj_ < 0.01, Cohen's *d* = 1.09), not in the sham group (*p*_adj_ = 1, Cohen's *d* = 0.28). Inter‐group comparison using unpaired *t*‐test was significant (*p*_adj_ < 0.01, Cohen's *d* = 1.32). Reduction in the trunc subscore between V0 and V1 showed a trend towards more improvement in the verum group, but was not significant after Bonferroni correction (*p*_adj_ = 0.122, Cohen's *d* = 0.70). In the sham group there was also no significant change of the trunc subscore (*p*_adj_ = 0.106, Cohen's *d* = 0.64).

For the secondary outcome parameters 8MW and PATA rate, there was a significant reduction between V0 and V1 in the verum group (*p*_adj_ < 0.05 with *r* = 0.76 for 8MW, and *p*_adj_ < 0.01 with Cohen's *d* = 1.31 for PATA rate), but not in the sham group (*p*_adj_ = 1 and *r* = 0.26 for 8MW and *p*_adj_ = 0.131 with Cohen's *d* = 0.61 for PATA rate). Intergroup comparison was not significant for these outcome measures (*p*_adj_ = 0.251 and *r* = 0.37 for 8MW and *p*_adj_ = 0.092, *r* = 0.94 for PATA rate).

For the secondary outcome parameter dynamic posturography, there was no significant improvement between V0 and V1 neither in the verum (*p*_adj_ = 1, *r* = 0.04), nor in the sham group (*p*_adj_ = 1, *r* = 0.07).

There was no significant change in the NHPT between V0 and V1, although there was a trend towards more improvement in the sham group that was not significant after correction for multiple testing (*p*_adj_ = 0.316_,_ *r* = 0.48 in the sham group versus *p*_adj_ = 1, *r* = 0.07 in the verum group).

## 4 DISCUSSION

In this randomized, double blind, sham‐controlled trial, we demonstrate the positive effects of cerebellar rTMS in combination with intensified symptomatic physiotherapy on ataxia severity in SCA patients. The strengths of this study include the use of neuro‐navigation for improved target localization, which is still rare in TMS studies,^11^ the genetically determined study cohort and application of additional symptomatic physiotherapy in both treatment groups. All assessments were performed by SARA‐trained and certified investigators. Despite the accelerated protocol, treatment was generally well tolerated with only minor adverse events that were mostly limited to immediate application of TMS pulses. Only one patient experienced mild headache that persisted between TMS sessions.

The effect of TMS on cerebellar ataxia has been positively reviewed in three publications.^11^, ^23^, ^26^ However, the reviewed studies used different stimulation protocols (excitatory and inhibitory) and study designs (from case reports to randomized controlled trials) and the positive effects were attributed to different aspects of the symptom ataxia. Wang and colleagues reviewed 8 qualitatively heterogeneous TMS studies in patients with mixed cerebellar ataxias (three of the reviewed studies included cerebellar strokes as cause of ataxia). Only four of the reviewed studies included the SARA as outcome measure. A meta‐analysis of these studies showed a mean reduction of 2.6 SARA points after TMS. Qui and colleagues reviewed 6 studies with cumulatively 157 SCA3 patients and a treatment duration between 2 and 4 weeks.^11^ The included studies partly overlapped with those from Wang and colleagues,^17^, ^18^ but the quality of the included studies was generally higher and focused on a more homogenous cohort. Their meta‐analysis showed a mean reduction of 1.59 SARA points, which seems to be a more realistic estimation of treatment effects. Notably, only one of the reviewed studies investigated an excitatory stimulation protocol.^22^ Recently, Shi and colleagues published the largest TMS study in SCA3 to date, with 120 patients.^20^ The study comprised of two treatment groups (one excitatory iTBS stimulation protocol and one inhibitory protocol) and a sham group. Treatment duration was 2 weeks (with 10 sessions on 2 × 5 consecutive days and 1200 pulses per session) without long‐term follow‐up visits. Interestingly, excitatory and inhibitory TMS both yielded significant reduction in ataxia severity as compared to the sham group (−1.5 SARA points in the inhibitory stimulation protocol and −1.9 SARA points in the iTBS group). Comparison of the treatment effects between the intervention groups was not significant.

Although treatment duration in our study was much shorter than the studies discussed above, the treatment effects on ataxia severity as measured by the SARA are at least comparable. The shorter treatment duration of our study protocol facilitates application in an outpatient setting and therefore increases availability to a larger patient population, which is encouraging considering the lack of effective treatment for these patients. On the other hand, the study duration may have been too short to observe therapeutic effects of intensified symptomatic physiotherapy alone. This is a possible explanation for the lack of significant improvement in all outcome measures in the sham group.

Although overall effects of TMS on ataxia severity seems to be stable across the cited studies, there is uncertainty about the domains of ataxia which best respond to treatment:

### 4.1 Trunc ataxia

The SARA items are weighted differently according to their resulting functional impairment. SARA items 3 (sitting) and 5–8 (nose‐finger test, finger chase, fast alternating hand movements, and heel shin slide) are rated on a scale of 0–4 points, SARA item 1 (gait) has 0–8 points, and items 2 (stance) and 4 (speech disturbance) have 0–6 points.^4^ The SARA trunc subscore (items 1–4) therefore incorporates a maximum of 24 points and the appendicular subscore (items 5–8) has a maximum of 16 points.

In our study, rTMS did not lead to significant improvement in the SARA trunc subscore neither in the sham, nor in the verum group. This subscore is biased towards gait and stance function, because these items contribute most points to the subscore and, at the same time, correlation between these items is usually high. Changes in item 3 usually appear only in later disease stages and become functionally relevant when patients are wheelchair bound. In these cases, Items 1 and 2 have reached a ceiling effect. None of the patients in our study were wheelchair bound, so item 3 potentially plays an inferior role in this analysis.

Whilst SARA item 1 mainly focuses on gait quality and dependency of assistive devices but neglects the time factor, time is the only measured variable during the 8MW test. This difference may easily explain the apparent discrepancy between the significant reduction in 8MW after rTMS, but no significant change in the SARA trunc subscore in this study. Gait speed is also included in the international cerebellar ataxia rating scale (ICARS), which may be an explanation for previously reported improvements in this subdomain.^11^, ^20^ ICARS was not included as outcome measure in our study, so comparison of study results is limited. Furthermore, our study did not show significant changes in objective measurement of postural control. Again, this finding is internally consistent with the SARA trunc subscore reported in our study, but with a discrepancy with the reported effects of rTMS on the ICARS gait and balance subscore.^11^, ^20^

### 4.2 Speech disturbance

The role of cerebellar rTMS on speech disturbance, that is also incorporated in the trunc subscore, cannot be entirely explained by this study. On the one hand, SARA item 4 is used to assess speech disturbance during free speech by articulation and comprehensiveness according to the investigator. This introduced a bias of subjectivity to the speech item. Furthermore, the speech item is the only item that is excluded from SARA certification, which may lead to a higher intra and inter‐rater variability. On the other hand, PATA rate is used to assess speech disturbance during a standardized syllable repetition task. Patients repeat PATA at maximum speed for 10 s. The investigator counts the mean number of repetitions from two consecutive trials. The measure is numeric and can therefore be considered more objective. Cerebellar rTMS led to significant improvement in the PATA rate in the verum group only, whereas the SARA trunc subscore (and SARA item 4) showed no significant change. Shi and colleagues also found a significant improvement in PATA rate after inhibitory rTMS and iTBS as compared to sham stimulation.^20^ However, the study did not include other speech assessments and SARA item 4 is not reported separately. Other studies either did not include speech disturbance as an outcome measure at all, or failed to show iits improvement.^17^, ^18^, ^21^, ^23^, ^27^ The discrepancy between the two speech assessments in our study may be explained by different mechanisms underlying free speech and the syllable repetition, but the exact effects remain to be explored in further studies using more suitable assessment strategies.^28^

### 4.3 Appendicular function

In our study, the SARA appendicular subscore significantly improved after rTMS, but not after sham stimulation. Consequently, the improvement in total SARA is mainly caused by changes in this subdomain. In contrast, NHPT did not show a significant improvement. This finding is in line with other studies.^11^, ^20^ The performance of NHPT can be highly heterogeneous even within a subject and likely to produce outliers, for example by dropping a peg. Despite improved appendicular motor function, patients could therefore apparently worsen in NHPT after accidentally dropping a peg. This may be a possible explanation for the discrepancy between NHPT and other ratings of appendicular function among the studies.

### 4.4 Limitations

Cerebellar ataxia is the common feature of all SCAs, but there is a pathophysiological heterogeneity within the disease group. Despite our genetically determined cohort, there may be different treatment effects between the SCAs that cannot be explored in this study and we cannot rule out that, for example a higher prevalence of polyneuropathy in one treatment arm could have influenced the results of this study. The small number of investigators and standardized training and certification increases reproducibility by reducing inter‐ and intrarater variability. On the other hand, the monocentric study design is a limitation of this study.

Due to the low prevalence, SCA patients frequently need to travel long distances for treatment and study‐participation. Patients in our study were recruited from all over Germany. However, this limits the willingness to undergo long‐term follow‐up examinations, which explains the overall low number of long‐term follow‐ups in this and previously reported TMS ataxia studies and made statistical analysis of the V2 visits in our study impossible. Furthermore, assessments at V0 and V1 were performed in immediate temporal connection to the intervention. V2 however, was unrelated to therapy and patients had the burden of travel just for an examination. In fact, travel duration and realted costs were the most frequently mentioned reasons for missing out on V2.

Whether longer follow‐up periods can be realized in future studies is questionable in view of the long and arduous journeys involved.

Mean SARA scores were slightly higher in the verum group, although not statistically significant. Still, this constellation may increase the potential benefit of a therapeutic intervention in the verum group compared to the sham group, which is a potential limitation of this study.

Increased scalp to surface distance causes a decrease in electric field strength.^29^ Despite inter‐individual differences, the deep location of the (atrophic) cerebellum and especially the vermis makes this region vulnerable to this effect. Furthermore, we cannot prove that the fixed stimulator output intensity is sufficiently high to induce therapeutic effects in the target locatilon. Therefore, we cannot guarantee that the full therapeutic dose has reached its target in all patients. Latest generation neuro‐navigation provides direct feedback if the distance between coil and target is too large. Application of this technology would likely increase therapeutic effects. The figure of eight coil used in this study is commonly used for cerebellar TMS in literature. The electric field induced by this kind of coils can reach the cerebellar surface, but cerebellar inhibition (CBI) can be produced more reliably using a double cone coil with a higher penetration depth.^30^ It is therefore possible that the effect of this and the other cited studies is based on a different mechanism than CBI, which remains to be explored by future studies.

## 5 CONCLUSION

The lack of medical treatment underlines the importance of symptomatic therapy in SCAs. In this randomized clinical trial, we demonstrate the positive effects of cerebellar rTMS on ataxia severity in these patients. Our results are in line with results from previous studies, but condensing treatment duration to only 5 days without reduction of treatment effects facilitates application and therefore broadens availability to larger patient populations. Longer duration of intensified physical therapy would likely have contributed to greater overall therapeutic effects. Future studies should therefore focus on the optimal combination of rTMS and physiotherapy to fully exploit the therapeutic potential.

## AUTHOR CONTRIBUTIONS

Conceptualization: Oliver Kaut. Methodology: Marcus Grobe‐Einsler, Oliver Kaut. Formal analysis Marcus Grobe‐Einsler, René Hurlemann. Investigation: Marcus Grobe‐Einsler, Friederike Bork, Aline Faikus. Writing—original draft preparation: Marcus Grobe‐Einsler. Writing – review and editing: Friederike Bork, Aline Faikus, René Hurlemann, Oliver Kaut. Supervision: Marcus Grobe‐Einsler, Oliver Kaut.

## CONFLICT OF INTEREST STATEMENT

MGE received research support from the German Ministry of Education and Research (BMBF) within the European Joint Program for Rare Diseases (EJP‐RD) 2021 Transnational Call for Rare Disease Research Projects (funding number 01GM2110), from the National Ataxia Foundation (NAF), and from Ataxia UK, and received consulting fees from Healthcare Manufaktur, Germany, all unrelated to this study. MGE is member of the European Reference Network for Rare Neurological Diseases (ERN‐RD). AF received travel support from CSL Behring, Ipsen, Ever Pharma and Indorsia, all unrelated to this project. AF and OK received research support from the German Parkinson's Association (Deutsche Parkinson Vereinigung), unrelated to this project.

## PATIENT CONSENT STATEMENT

All enrolled subjects gave written informed consent for participation.

## Supporting information


Figure S1.

Figure S2.

Appendix S1.

## Data Availability

The data that support the findings of this study are available from the corresponding author upon reasonable request.
