# Supplementary figures and images for: Effects of cerebellar repetitive transcranial magnetic stimulation plus physiotherapy in spinocerebellar ataxias – A randomized clinical trial
Source: CNS Neurosci Ther. 2024 Jun 18;30(6):e14797. doi: 10.1111/cns.14797 (PMC11183922; doi:10.1111/cns.14797)

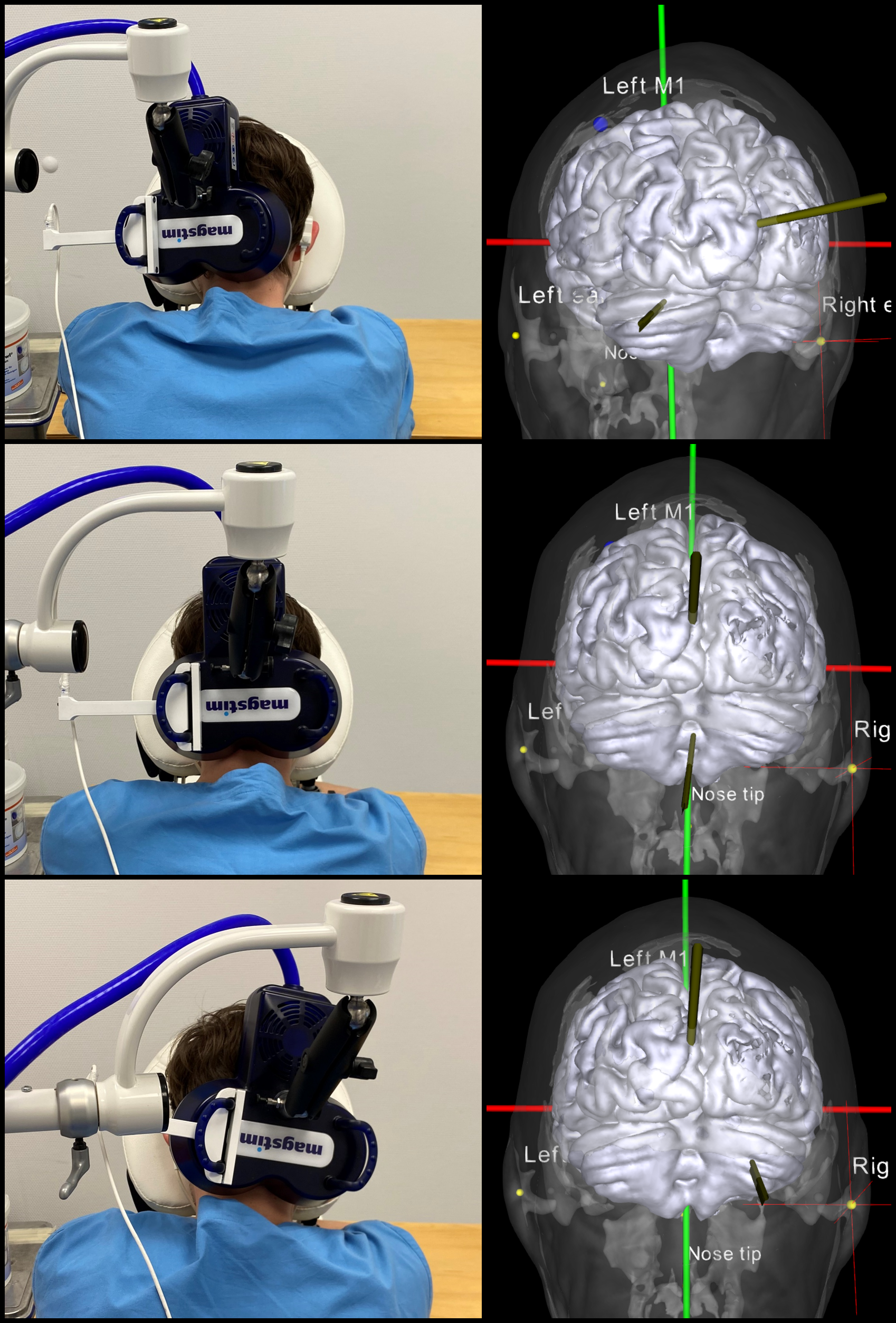

Supplement: Supplementary file 1 — Figure S1. [file CNS-30-e14797-s001.png]

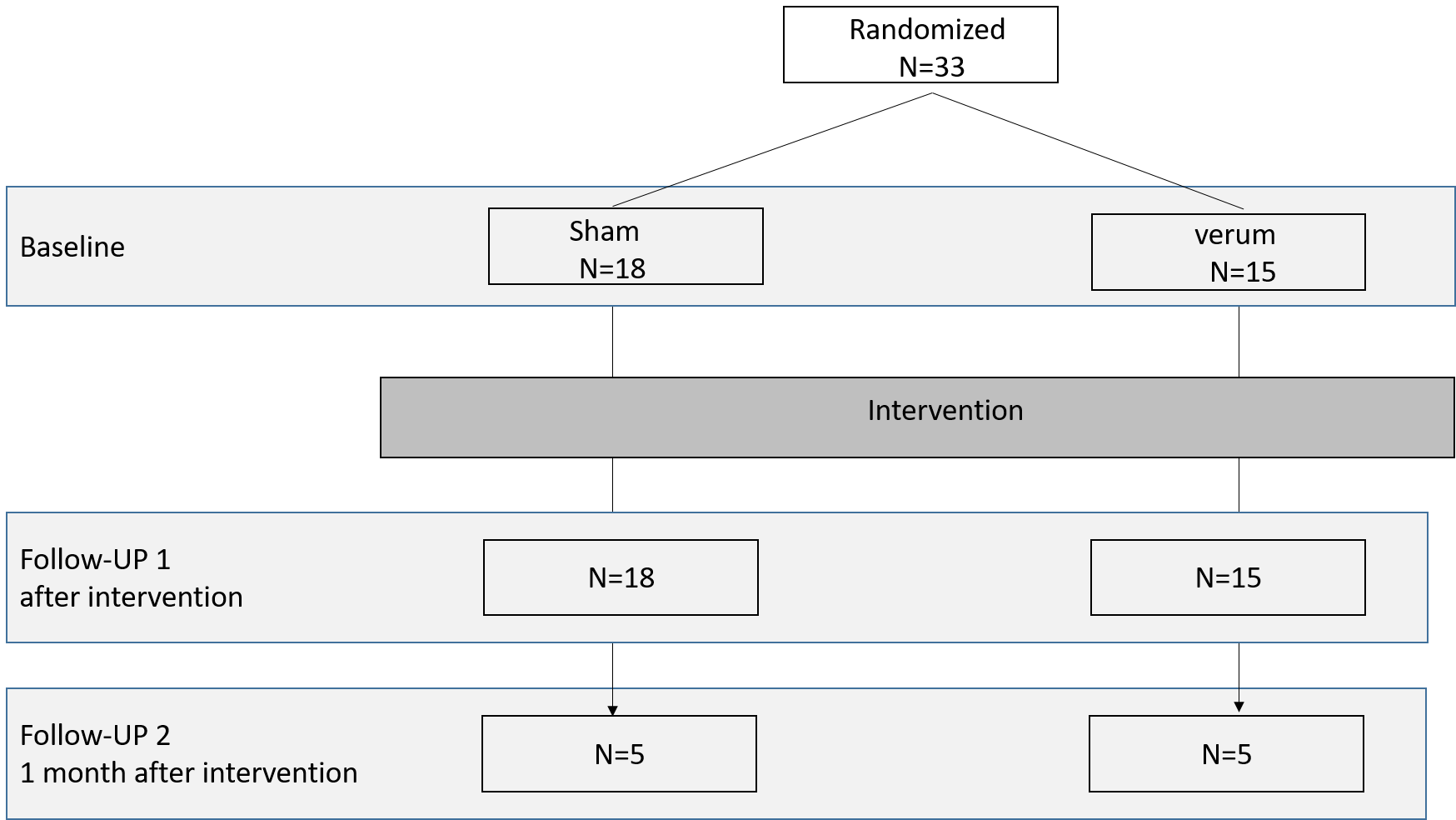

Supplement: Supplementary file 2 — Figure S2. [file CNS-30-e14797-s003.png]
