# Supplementary material for: Effects of cerebellar repetitive transcranial magnetic stimulation plus physiotherapy in spinocerebellar ataxias – A randomized clinical trial
Source: CNS Neurosci Ther. 2024 Jun 18;30(6):e14797. doi: 10.1111/cns.14797 (PMC11183922; doi:10.1111/cns.14797)
Supplement: Supplementary file 3 — Appendix S1. [file CNS-30-e14797-s002.docx]

**Supplementary FIG 1:** Images of the three stimulations sites from top down: left cerebellar hemisphere, vermis and right cerebellar hemisphere.

**Supplementary FIG 2:** study flow-chart
